# Barriers to Care for Pediatric Patients with Obesity

**DOI:** 10.3390/life14070884

**Published:** 2024-07-17

**Authors:** Sreevidya Bodepudi, Malikiya Hinds, Kayla Northam, Noreen A. Reilly-Harrington, Fatima Cody Stanford

**Affiliations:** 1 MGH Weight Center, Massachusetts General Hospital, Boston, MA 02114, USA; knortham@mgb.org (K.N.); nharrington11@mgh.harvard.edu (N.A.R.-H.); 2 Harvard Medical School, Boston, MA 02115, USA; 3 MGH Weight Center, Massachusetts General Hospital, Department of Medicine-Division of Endocrinology-Neuroendocrine, Department of Pediatrics-Division of Endocrinology, Nutrition Obesity Research Center at Harvard (NORCH), Boston, MA 02114, USA

**Keywords:** obesity, pediatric obesity, barriers, stigma, health equity

## Abstract

This review article emphasizes the challenges pediatric patients face during obesity treatment. Prior research has been compartmentalized, acknowledging that stigma, the ability to implement lifestyle changes, social health determinants, and healthcare accessibility are considerable impediments for obese children. These issues emerge at various levels, including the individual or family, the community and school, and even national policy. This suggests the need for a more comprehensive, team-based approach to tackle pediatric obesity. Understanding these barriers is the first step toward creating effective strategies and solutions to overcome these challenges.

## 1. Introduction

Childhood obesity is a rapidly developing problem on a global scale. In the years of 2017–2020, 19.7% of children and adolescents were affected by obesity, which accounts for 14.7 million children [1]. Obesity rates are especially high among Hispanic children (26.2%) and non-Hispanic Black children (24.8%) [1]. If obesity continues to expand at this rate, 9% of all preschoolers will develop overweight or obesity [2]. In the United States, Brazil, and China, pediatric obesity has been increasing at higher rates when compared to adults [2]. This increases the risk of children developing chronic medical conditions such as type 2 diabetes, sleep apnea, joint pain, and mental health conditions [1]. When childhood obesity is not treated, there is a greater risk of these populations continuing to have obesity in adulthood, increasing the risk for disease and disability long term [2].

With all the sequential effects of obesity, it is becoming increasingly important to prevent and treat obesity aggressively. In our current healthcare climate, while this has been identified as a rising emergency, numerous barriers stand in the way of being able to treat childhood obesity adequately. This paper will highlight the various obstacles patients face in treating obesity.

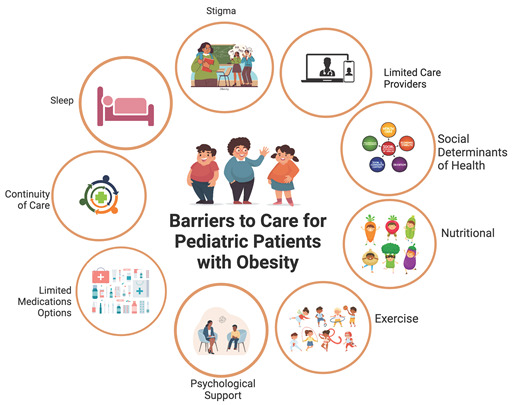



### 1.1. Stigma

Stigma against patients with obesity continues to prevail as a belief that shame will motivate people to lose weight [3,4]. However, it has been identified that stigma propagates obesity by contributing to harmful behaviors, including social isolation and binge eating [3,4]. Additionally, patients who are stigmatized often avoid seeking medical care, exacerbating other medical conditions [4]. Caucasian women of low income are the most vulnerable to weight stigmatization [5]. Weight stigma can develop in children with overweight or obesity as early as 3 years old [4,6]. Recognizing that stigma arises from multiple sources, patients are consistently exposed. Many patients who experience stigma are stereotyped as lazy, unmotivated, or lacking in willpower and discipline [4]. While many sources of stigma exist, parents continue to play a role in this perpetuation. In a survey of adolescents attending weight-loss camps, 37% of adolescents reported being teased or bullied about their weight by a parent, and bullying and teasing occur regardless of race, socioeconomic status, and academic achievement [4,5]. Parents are also likely to exhibit implicit bias. Weight bias can lead to prejudice, social rejection, and discrimination [3,4].

On the other hand, overweight and obesity may be underdiagnosed by parents. A self-reported survey conducted in Italy surveyed 649 adolescents with obesity and 455 caregivers. This survey found that compared to adolescents living with obesity, their caregivers were less likely to identify their child has overweight or obesity and were more likely to believe their child would lose excess weight with age [7]. If caregivers do not identify overweight or obesity as a concern, there is a higher risk of delayed diagnosis and treatment [7].

In children, there are higher rates of weight-based victimization, teasing, and bullying, and this only increases with a child’s body mass index (BMI) percentile [3,4], recognizing that BMI is a ratio of weight (in kilograms) to height (meters squared) [8]. This is one of the most frequent forms of peer harassment in school systems [3,4]. Children and adolescents with severe obesity who have experienced such stigma experienced worse quality-of-life scores compared to matched children who were diagnosed with cancer [6].

Stigma exists and persists in our larger communities. Research has shown that teachers have lower expectations of students with obesity compared to students without obesity. When compared to test scores, teachers’ assessments of children with obesity were constantly lower, showing that these scores were not representative of a student’s capacity [4]. In an Australian study, it was found that health and physical education teachers reported stronger implicit and explicit bias due to a lack of concrete inclusivity training [5]. This stigma impedes students from participation in physical activity while also causing social barriers and impacting psychological well-being [5].

Beyond the daily environments of school and home, weight stigma is perpetuated through the media. Visually slim children are often portrayed as the main characters and are seen as kind, popular, and attractive [4]. On the other hand, characters with larger bodies are often depicted as unpopular, aggressive, evil, and unhealthy [4]. One analysis revealed that television shows targeted toward adolescents are 10% more likely to incorporate weight-stigmatizing content, and over two-thirds of images in US media reports of obesity contain weight stigma [4,9]. Shows focused on weight loss, such as The Biggest Loser, also reinforce stereotypes, as it was found that viewers disliked individuals with excess weight more after viewing the first episode [5]. On social media and video-sharing websites like YouTube, more verbal attacks were posted anonymously in the comments section of videos including individuals with overweight or obesity [5].

Health care professionals also perpetuate stigma. Physicians, nurses, dieticians, psychologists, and medical trainees self-report bias and prejudice toward patients with obesity [4]. A 12-year study found that even obesity medicine specialists demonstrated explicit bias [5]. A different study found medical students to display greater explicit bias against patients with obesity compared to racial minorities, sexual minorities, and people of lower economic backgrounds [10]. The stereotypes that patients with obesity are nonadherent, lazy, and lack self-control are held by such providers [4,9]. This diminishes the quality of care provided, as providers spend less time with patients and are less likely to complete preventive health screenings [4]. Hence, medical schools and other educational facilities can reduce bias by improving education efforts about weight stigma and prevalence while also combating stereotypes while teaching [5]. Additionally, offices are not always equipped with appropriately sized equipment, including examination tables and gown sizes [4]. These biases contribute to patient hesitancy in seeking care.

All this stigmatizing behavior may worsen obesity by reinforcing unhealthy behaviors [4]. Adolescents who experience bullying are more likely to develop depression, anxiety, self-harm behaviors, low self-esteem, poor body image, and substance use disorder [4,10]. Reports have shown that adolescents with obesity are two times more likely to contemplate and attempt suicide compared to adolescents without obesity [4]. There is also an increase in social isolation. A study found that two-thirds of 9- to 11-year-old children who were perceived as having excess weight believed that losing weight would lead to having more friends [4]. These beliefs help perpetuate unhealthy eating behaviors and more emotional and binge eating [4,10]. These populations also felt less confident participating in physical activity [4]. A combination of these factors can lead to worsening obesity [4].

Obesity stigma has also been perpetuated by healthcare systems and insurance companies. While obesity was formally recognized as an epidemic in 1998, this has been slowly accepted by the medical community [11]. The American Medical Association did not recognize obesity as a complex chronic disease until 2013 [12]. Currently, while clinical guidelines incorporate the use of lifestyle changes in conjunction with pharmacotherapy and/or bariatric surgery, Medicare excludes drug therapy for obesity [13]. Medicaid coverage varies widely between states. As of November 2023, only 16 states provide coverage for anti-obesity medications via Medicaid [14]. When insurance companies fail to recognize obesity as a chronic disease and cover appropriate treatments, this limits patient access to guideline-directed care.

### 1.2. Limited Care Providers

Even when patients are amenable to treatment, there are several barriers to providing this care. With so many aspects of health to address, screening for obesity at well-child checks is not consistently completed by primary care providers [15]. Several studies have shown that the identification of overweight/obesity in primary care clinics is low, with less than 30% of children with overweight being identified [16]. Many providers share a lack of training in obesity management as a barrier to providing care [15]. As of 2013, only one in four of a thousand graduating pediatric residents felt that their management was effective [15]. Many providers tied their expectations for counseling outcomes to their initiative to provide counseling [17]. While this confidence is increasing, a gap in education remains, and many providers still feel that their counseling could be more effective [15,17].

Primary care physicians generally self-report a low ability to counsel parents about obesity and engaging in behavioral changes [16,18]. A survey of pediatric providers in Louisiana reported that while 88% of pediatric providers screen for obesity, only 7% followed guidelines for referring patients to weight management resources [19]. In addition, only 6 of 57 providers incorporated all recommended components of pediatric obesity management into their care plan [19]. Research conducted shows a dichotomy between a strong desire to address obesity while having low confidence in the effectiveness of providing care [20]. In a study collecting cross-sectional survey data from 123 providers from 23 practices in North Carolina, 43.1% of providers had received additional training in obesity. This group of providers reported higher outcome expectations for increasing outdoor activity, decreasing sugar-sweetened beverage or juice intake, and reducing screen time [17]. These providers also reported increased confidence that providing lifestyle counseling would result in a behavior change [17]. With a limited number of tertiary treatment sites available to provide patient care, improved educational efforts need to be focused on primary care physicians [16]. However, providers adopt better screening practices when provided with a referral source [16].

In addition to a knowledge gap, primary care physicians reported limited care time with patients, inadequate reimbursement for obesity-related services, limited clinic resources, and limited community resources as additional barriers [15,16,19,21]. Many primary care clinics cannot integrate the services of a registered dietician to support physicians in providing lifestyle counseling [3]. Even if such resources exist, fewer dietitians elect to complete additional pediatrics training, limiting the available workforce [21]. There is also a mismatch in clinics’ understanding of how to bill for dietician services so that these tools can be better supported in an outpatient setting [18]. While motivational interviewing can help make behavioral changes, many providers need additional training or staff to deliver these interventions [20]. Without these supportive elements, providers described not feeling confident in providing comprehensive patient care [17]. In the Louisiana study referenced above, while all providers recognized the importance of discussing obesity with patients, 23% of providers felt behavioral management of pediatric obesity should not be completed in a primary care setting without these other support elements [6]. This lack of support structure makes it difficult to provide comprehensive care.

On a systems level, in a study completed in North Carolina in 2013, only 3 of 23 practices could calculate BMI through the electronic medical record [17]. Currently, while there are other methods to quantify obesity and the severity of the disease, BMI continues to be the most prevalent as it is easy to calculate [22]. Many providers also recognize that with limited time, efficient use of the electronic medical record would be more helpful. In one study, two-thirds of providers indicated that a Best Practice Advisory notice for patients with BMI greater than the 85th percentile and a smart set or algorithm to help determine the next steps in management would be helpful [16]. Using technological advances more effectively provides more efficient and adequate care.

### 1.3. Barriers to Lifestyle Changes

At the foundation of all treatment for obesity remains intensive lifestyle changes. Efforts in lifestyle changes primarily focus on nutrition and dietary changes in combination with increasing exercise and activity levels. However, many blockades exist to be able to make such changes. In the following sections, such barriers will be reviewed as they pertain to a specific lifestyle change (i.e., nutrition, physical activity, etc.). On a broader scale, adolescents reported that it is difficult to change established barriers. In a study of 19 adolescents in Canada, one participant stated “I know I wouldn’t make healthy food myself…If I made my own food it would just be a bath of junk food” [23]. The same participant shared that making healthier food choices is easier when one likes to have a choice over food intake [23]. Additionally, a lack of control over mental health was identified as another barrier. Adolescents reported difficulty addressing anxiety and depression, especially when these diagnoses were not acknowledged by their parents. Untreated anxiety and depression can lead to decreased energy levels and avoidance of social interactions, which can lead to decreased activity [23]. Increased depressive symptoms can also lead to increased emotional eating [24].

### 1.4. Social Determinants of Health

Prior to addressing specific lifestyle changes, it is important to recognize how social determinants of health affect these behavioral interventions. Hahn defines social determinants of health (SODH) as encompassing the structures and elements of society and the social assets and risks for health that these structures manage, distribute, and limit, leading to health outcomes and shifts in health patterns and distributions [25]. It is important to assess the implications of social determinants of health on weight outcomes.

Studies conducted by Javed and colleagues utilized data from the 2013 to 2017 National Health Interview Survey involving 161,795 adults to investigate the impact of SDOH on overweight and obesity. The study aggregated 38 SDOH into a cumulative score, divided into quartiles to indicate SDOH burden levels. The study found a clear correlation between increasing SDOH burden and higher rates of overweight and obesity, with significant disparities across age, sex, and race/ethnicity. After adjusting for relevant factors, the highest SDOH quartile was associated with a substantially elevated prevalence of overweight and obesity compared to the lowest quartile. The study concluded that a status of social disadvantage over time, as indicated by a greater burden of social determinants of health, was linked to higher chances of obesity, regardless of clinical and demographic variables [26].

Social determinants of health can also impact one’s level of physical activity, which has implications on healthy weight. Trends in the built environment increasingly limit opportunities for physical activity through changes in urban landscape and design, poor neighborhood walkability, and limited options for public transport [27,28,29]. Findings also show that occupation largely dictates energy expenditure [30]. Data also show that reduced workplace activity, a commute involving a vehicle, and computer-based work tasks have contributed to worsened weight outcomes [31]. An increase in the sedentary lifestyle in both the workplace and everyday life activities, including watching television, video games, and screen time has become more prevalent and appears to promote the overconsumption of food [32]. In culmination, these trends lead to an overall reduction in energy expenditure [33].

Studies show that the global food system has increasingly favored highly processed, energy-dense, and nutrient-poor foods known as the “Western diet”, characterized by high sugar and fat and low fiber levels. This shift has been endorsed by targeted marketing strategies and economic factors that make processed foods cheaper and fresh produce more expensive, thus discouraging healthy eating behaviors [33]. These global trends have contributed to a significant global rise in caloric intake, mainly from carbohydrates and sugary drinks, which is amplified by larger meal sizes and frequent snacking. This dietary shift has coincided with a dramatic increase in obesity rates, particularly evident in the US, where obesity more than doubled over a few decades [33].

Additionally, food access within the built environment can also be linked to detrimental health outcomes. When proximity to supermarkets and healthier food options is limited, it adds an extra challenge for communities to access food and have sufficient nutrition. Subsequently, patients living in food deserts are more likely to develop obesity and diabetes [34]. Food deserts can lead to food insecurity when citizens in these areas rely on high-calorie low-cost foods available at convenience stores and fast food restaurants [35,36]. As access is a barrier, food-insecure homes generally store more obesity-promoting foods, such as microwavable frozen meals [35]. Food insecurity can lead to chronic stress, which can affect cognitive development and also increase the risk of obesity [35]. Many children become more worried, angry, or frustrated and live with family isolation and sadness. Some children will try to cope by distracting themselves and increasing their tolerance of the family food situation. This may lead to poor-quality diets. With excess stress, cortisol levels increase and result in increased caloric intake [35]. Patients who experience food insecurity are more likely to develop disordered or binge-type eating patterns [35].

Global trends show similar dietary shifts began in high-income countries in the 1970s and 1980s, with middle and low-income countries following as their economies grew and urbanization increased. These changes have disproportionately affected rural, poor, and minority populations, who are more exposed to the environmental determinants of obesity. Recent data indicate that rural areas now have higher rates of overweight and obesity than urban areas, even in low- and middle-income countries [33].

Additionally, poverty plays a complex role in obesity, with rates being the highest among the poor in high-income countries. In contrast, in low- and middle-income countries, obesity initially affects wealthier demographics before shifting to the poorest as economies develop. More studies are needed to clarify further poverty stratification and its interplay with obesity [33]. Data also support the influence of ethnic and racial disparities on obesity rates, with higher prevalences observed in African Americans, Native Americans, and Pacific Islanders compared to Asian Americans in the US. Meanwhile, migrants to high-income countries initially arrive healthier but often experience weight gain over time that exceeds native population levels due to influences of socioeconomic, sociocultural, and genetic factors [33].

When considering the relationship between obesity and competing life necessities, a study by Pope and colleagues shows significant differences between various barriers and facilitators that promote healthy weight among school-aged children in rural environments [37]. Data show that barriers at the family level, such as limited financial means and competing priorities, significantly contributed to the prevalence of unhealthy weight behaviors in these demographics. A study analyzing data among Midwest hospitals found that every additional social risk that was reported during a well-child visit increased the odds of multiple emergency room visits and hospitalizations in the next year [38].

Meanwhile, the positive influence of parental role modeling via healthy eating habits and exercise was recognized as promoting healthy weight behaviors. On the organizational front, childcare providers and community stakeholders highlighted challenges like insufficient funding and low parental engagement in health promotion initiatives. Childcare providers noted adherence to strict nutrition and physical activity guidelines but voiced concerns about inconsistent reinforcement of these messages at home. Organizational facilitators included providing healthy meals at no charge in childcare programs and health promotion initiatives conducted by community organizations. At the community level, obstacles such as inadequate public transportation and limited access to nutritious food outlets and resources for physical activity were observed. However, existing resources like parks were seen as assets. It is important to continue discussions regarding potential child obesity prevention interventions, as this study emphasized the importance of building community trust, shifting focus to wellness rather than solely obesity prevention, forging community partnerships, and making the most of available community assets in the promotion of health weight behaviors [37].

A study conducted by Yusuf and colleagues found that SDOH is representative of significant markers of overweight or obesity in children [39]. Approximately 30.6 million children participated in the survey, revealing that 9.5 million (31.0%) were classified as either overweight or obese. The likelihood of obesity was higher among non-Hispanic Black and Hispanic children, with prevalence ratios (PR) of 1.53 (95% CI = 1.01–2.31) and 1.50 (95% CI = 1.18–1.90), respectively. Overweight tended to be more prevalent in younger children, those with single parents, and those residing in neighborhoods lacking amenities. Factors such as parental college education, private health insurance coverage, female gender, and a language spoken at home other than Spanish were found to be protective against overweight or obesity [39].

These SDOH factors were found to be protective due to associated families’ access to resources and the opportunity to attain and maintain healthier physical and mental lifestyles [20]. This study and similar analyses need to be repeated on a larger scale to assess the wider implications of these factors on racial/ethnic disparities, population-based policy development, funding allocation, and the prevention of overweight and obesity in childhood and, subsequently, adulthood [20].

While the importance of addressing SDOH factors has been clearly identified, screening for such factors remains low. The Centers for Medicare and Medicaid Services Accountable Health Community Model identifies five basic social needs, including housing instability, utility needs, transportation needs, and experience with interpersonal violence. However, only 25% of hospitals and 16% of physician practices report screening for these needs [38]. While guidelines have been created to properly screen for SDOH, the availability of resources, workflow, and staff buy-in are barriers to implementation [38].

### 1.5. Nutritional

On a family level, there are nutritional and dietary contributions to pediatric obesity, which can also be barriers to change. Consuming high quantities of unhealthy food is a major contributor to obesogenic behaviors [40]. Children and adolescents are more likely to consume insufficient amounts of whole grains and unprocessed foods. This is especially true in those who skip breakfast [41]. When combined with diets focused on calorie restrictions for the treatment of obesity, this can contribute to nutrient deficiencies, which can impair normal growth and development [40]. Children also have increased exposure to caffeine compared to the past; one study found that 98% of children between the ages of 2 and 3 consumed caffeine during a 24 h period [42]. Not only do many caffeinated drinks have high sugar content, but the caffeine intake also correlates with an increased intake of calories, as well as less protein, fiber, and dairy [42]. In a study of high-risk Mexican American children, children ingested 121 more calories on days that caffeine was consumed, and children consumed caffeine on 29% of recall days during this study. Over the course of 12 months, this equates to 10,527 additional calories [42].

As sugar-sweetened beverages have become so widely available and affordable, they have added fuel to the rising rates of pediatric obesity. Sugar-sweetened beverages constitute the largest portion of caloric intake among children [43]. The excessive intake of such beverages leads to an increase in BMI, waist circumference, and body fat percentage [44]. One study showed that children who drank sugar-sweetened beverages between 1.5 and 4.5 years old were 2.4 times more likely to be overweight at age 4.5. Another study found that children 2–5 years old who consumed more than one sugar-sweetened beverage a day were more likely to develop overweight or obesity over the next 2 years [43]. Additionally, the consumption of these beverages is more likely to lead to type 2 diabetes, hypertension, hyperlipidemia, and cardiovascular disease [43]. There has been some research into understanding the intake of excessive sugar-sweetened beverages leading to a sugar addiction, perpetuating this cycle [43].

In addition to sugar-sweetened beverages, the availability of ultra-processed foods (UPF) has escalated. Research shows that foods with a high glycemic index may cause more weight gain and adverse metabolic effects in women than in men, explaining the stronger link between ultra-processed foods and obesity in women. For men, significant weight gain from ultra-processed foods occurs only at very high intake levels. Ultra-processed foods contribute to weight gain by displacing nutrient-dense foods, encouraging overeating due to their convenience and aggressive marketing, and reducing satiety. Their impact on the gut microbiome also affects energy balance and lipid accumulation, influencing obesity [45]. Kevin Hall and fellow investigators performed a study that investigated 20 inpatient adults who were exposed to ultra-processed versus unprocessed diets for 14 days each, in random order. The ultra-processed diet resulted in an increase in desired energy intake by roughly 500 kilocalories a day and led to weight gain, despite being matched to the unprocessed diet for presented calories, sugar, fat, sodium, fiber, and macronutrients [46].

Further analyses of the association between UPF consumption and the incidence of obesity were conducted on a sample from nine European countries in the European Prospective Investigation into Cancer and Nutrition (EPIC) study [29]. After a median follow-up of 5 years, those who consumed an energy-adjusted average of 686 g a day of UPF (the highest quintile) had a 15% increased risk of developing overweight or obesity compared to those who consumed an energy-adjusted average of 176 g a day of UPF (the lowest quintile). Among those with overweight at baseline, there was a 16% increased risk of obesity in the highest versus lowest quintile. This study also found that for every one standard deviation increase in UPF consumption, there was a 0.12 kg increase in weight over 5 years. Like the cross-sectional studies in this review, the cohort studies consistently showed an increased risk of obesity with higher intakes of UPF [47].

Regarding cost, healthier dietary patterns, such as the Mediterranean diet, are sometimes more expensive [48]. Retail prices are significantly influenced by various players in the food supply chain [48]. Extending the shelf life of products reduces the risk of foodborne illnesses and decreases the tendency to overeat to avoid food waste, which is linked to overweight and obesity [48]. Longer shelf life also aids in better meal planning, reducing unplanned eating and improving energy compensation [48]. Moreover, making nutrient-dense, fiber-rich, low-energy foods more available (e.g., canned, frozen, dried fruits and vegetables, legumes, and grain products) supports weight management [48].

In addition to food quality, food timing is an essential component to consider in childhood obesity. Children who skip breakfast had a 43% greater risk of developing overweight/obesity compared to those who eat breakfast [41]. The most common reasons for skipping breakfast included sleeping late in the morning and a lack of appetite in the morning [41]. A proposed mechanism for this is that those who consume breakfast regularly experience greater thermogenesis than those who skip breakfast [41]. Another potential mechanism discussed is how skipping breakfast can cause circadian misalignment, especially in children and adolescents [49]. The children who skipped breakfast generally consumed less protein compared to breakfast consumers [41].

Many children rely on school breakfast and lunch programs for regular meals. Updated nutrition standards for school meals were introduced in 2012–2013 [50]. A study analyzing healthy eating index scores found that the updated standards significantly improved the nutritional quality of meals and were more consistent with the Dietary Guidelines for Americans [50]. However, only 37% of eligible students participated in free or reduced-price meals for breakfast, and 78% participated in lunch programs [51]. The reasons for decreased participation include meal palatability, diversity of menu options, and short length of meal periods. Breakfast options are generally offered before school starts, which may not be accessible for students who arrive by bus or cannot arrive at school early. Some schools have other food options, such as vending machines, which may be more appealing to students [51].

Most nutritional-based strategies utilize a “one-size-fits-all” model, which does not account for differences between children [40]. This can make it harder for children and families to adhere to dietary changes when these models are difficult to incorporate. These educational barriers start as early as infancy, as dietary counseling during the first 1000 days of a child’s life can have a significant impact and reduce the risk of developing obesity in the future [52]. While parents dictate where and when meals happen, as children age, it is important to involve them in the conversation [52]. When children focus on health and fitness rather than weight, this helps with developing healthy behaviors [52]. A lack of motivation to address obesity was another common barrier reported [16]. Providers felt that if parents did not recognize their child as having overweight or obesity, motivating parents to engage in treatment was difficult [16].

When examining childhood obesity, there is a range of educational knowledge that exists among parents, which can significantly impact the nutrition status of the entire family. A study of 261 students in Spain was conducted to examine the efficacy of an educational intervention. Parents of children aged 3–4 years old were randomized into a control group or the intervention group receiving nutritional education. After the first year of intervention, the joint prevalence of overweight and obesity increased in the control group from 12.2% to 20.1%, while the prevalence of overweight and obesity remained stable in the intervention group. The BMI in the intervention group significantly decreased by 0.10 compared to the control group, in which the BMI decreased by 0.01 [53]. A different study of 287 preschool children in Spain examined parental healthy eating attitudes and knowledge of nutrition. They developed a survey to identify a parent’s nutrition understanding and healthy eating attitudes and the potential relationship to their child’s intake and diet. This study found a positive association of parental healthy eating attitudes with nutrition adequacy and diet quality [54].

Positive parental role modeling can help facilitate healthy weight behaviors [21,37,55]. Children noted that eating habits are influenced by the family, as food availability within the home is generally dictated by parents [23]. However, if healthy behaviors are not modeled, that can also negatively impact a child’s behavior. Adolescents reported that expectations set by parents or peers can lead to pressure to eat unhealthy foods [23]. Additionally, when family members disagree about the lifestyle changes that must occur, this can lead to inconsistent child-feeding habits [55,56]. Many parents shared that a lack of nutritional knowledge and limited cooking skills were a barrier [29]. Facilitators of an organization in North Carolina created health promotion initiatives in which children could try new foods and grow fruits and vegetables in the garden [37]. Creating opportunities for children to be exposed to various foods and cultivating early curiosity can foster interest in diversifying consumption. Feeding habits are developed at a young age. Children with obesity tended to be breastfed for the shortest period and introduced to cow’s milk at the earliest [57]. They also tended to eat similar diets to the rest of the family early, with the latest introduction of vegetables, fruits, and meat products [57].

In addition to parental involvement, peers can also influence eating habits. Adolescents reported feeling pressured to consume unhealthy foods in social gatherings. Others reported being bullied for eating healthy foods [23].

When considering family-based changes, cultural backgrounds need to be considered. However, some cultural backgrounds can create further barriers. Many women continue to serve as the primary caretakers and struggle to balance traditional maternal roles and prepare meals for the family while balancing working life [55,56]. Many cultures also encourage patients to continue to “finish their plate” (Somali and Latino parents) or eat until they are “stuffed” (Hmong parents) [56]. Some cultures also follow restrictive patterns. A study showed that Chinese mothers closely monitored food intake, restricting unhealthy foods and mandating scheduled mealtimes [56]. This can lead to children struggling to learn how to make appropriate food choices and listen to hunger cues as they transition into adulthood. Lastly, many ethnic groups share that a “chubby” baby is a sign of a healthy baby, with some parents supplementing breast milk with formula and thickening formula with cereal [56]. Some of these cultural conceptions prove to be detrimental to long-term health outcomes.

From a school-based intervention scale, the limited number of health promotion programs serve as another barrier [37]. The limited time for meals also contributes to childhood obesity [55]. Children with obesity or overweight tend to eat quickly due to time constraints at school [55]. Schools elect to work with certain food service corporations, which can limit the involvement that schools can have in addressing nutrition [58]. School-based programs require buy-in from teachers and students, and limited engagement by either party will also limit the effectiveness of such programs [58]. An Australian study revealed that parents who were provided counseling on lifestyle changes from pediatricians needed more confidence in school-based interventions in weight monitoring and management [29].

On a community scale, social media constantly influences children [59]. Social media and gaming platforms create constant exposure ingrained into daily activity [60]. Children developing certain preferences for a particular food or brand are more likely to cultivate these preferences lifelong [59]. They are also more likely to perceive marketing as factual [60]. When this marketing includes celebrity endorsements, cartoon characters, or collectibles, this strengthens that influence, and these products become engrained preferences for children [59].

Over the past several years, the increased density of food outlets also makes this food more accessible to children [61]. A study conducted in the UK states that the closer fast food restaurants are to the school (within 400–800 m), the more detrimental this is for BMI [61]. For exposure within 1600 m, more children were seen to have an increase in BMI with lower emotional regulation [61].

Packaging plays a crucial role in influencing consumer behavior, especially when it involves “health” claims and optimized in-store presentations of branded ultra-processed foods (UPFs) in order to boost purchasing and utilization [48]. Misleading health labels, such as branding UPF items as organic, increase sales by falsely associating them with wholesomeness and health benefits [48]. This issue is not unique to UPFs, as consumers generally prefer products with organic labels across all food groups due to environmental and social responsibility concerns [48]. Health claims on packaging can overshadow actual nutritional information. Additionally, well-recognized food brands, often producers of UPFs, utilize targeted and aggressive marketing strategies to build trust and favorable perceptions of their products [48].

On a larger policy review, food insecurity continues to be a factor, leading to under and overnutrition [62]. While large associations cannot be drawn yet, there seems to be some data showing that the timing of food insecurity in a child’s life can affect future increases in BMI [62]. Limited funding for community-level programs continues to be a barrier [62].

Hunger and satiety signals are generated in regions of the brain that are not associated with conscious experience [63]. If irresponsibility were the cause of obesity, we would expect to see evidence of a general decline in personal responsibility. However, studies indicate otherwise. Research on laboratory animals shows that access to high-sugar, high-fat diets results in significant weight gain and poor health, even when healthier options are available. Humans are similarly affected by their environments. Factors like larger portion sizes, increased sugar intake, and the availability of calorie-dense foods contribute to weight gain. The modern food environment disrupts the body’s regulation of hunger and satiety, with sugar-sweetened beverages being a major contributor to increased calorie intake. Some studies suggest that certain foods can trigger addictive behaviors [63].

With the increased food access to ultra-processed foods and sugar-sweetened beverages by the food industry, the built environment for children has become more obesogenic [64]. The food industry utilizes aggressive marketing strategies to attract children and build brand loyalty, which can lead to the long-term consumption of these products [64]. The next focus is to integrate this evidence into public policy efforts to support the creation of healthy food environments and mitigate the influence of the food industry [64]. This step is essential to remove the ownership of disease from patients.

### 1.6. Exercise

On a personal and family basis, many children report time limitations (e.g., homework), personal barriers (e.g., interest), and environmental barriers (e.g., restricted access to equipment) as barriers to being more active. Some described feeling embarrassed or judged if unfamiliar with a sport or certain equipment [23]. Females tend to report higher levels of body consciousness, making playing sports more challenging [65]. Children also tend to model adults, and parent’s support and active involvement lead to increased activity levels [65]. One adolescent in a survey of 19 adolescents in Canada shared that he would be more likely to go to the gym if someone asked him to join versus being told to go without any company [23]. From the same survey, another adolescent noted that her peers are not physically active, so she does not feel motivated to be active [23]. These children also noticed less access to convenient places to be active or less gym equipment readily available as barriers [65].

Additionally, screen time has increased mindless eating with exposure to high-calorie and low-nutrient food and beverages [32]. Epidemiologic studies also show that children with excess screen media exposure consume fewer fruits and vegetables [32]. This often leads to overeating and less listening to hunger signaling between ghrelin and leptin. This also spills into sleep quality, as children sleep later and less [32]. In the previously referenced survey of adolescents from Canada, adolescents shared that they did not realize how much time they spent being sedentary. They described that while playing video games, they would lose track of time and have a hard time stopping [23].

Skipping breakfast was previously discussed from a nutritional standpoint, but also has effects from a physical activity perspective. Adolescents who skipped breakfast were more likely to have lower levels of physical activity throughout the day [66]. Additionally, male adolescents who regularly consume breakfast had higher scores in the 20 m dash, standing long jump, and shuttle compared to those who did not eat breakfast or ate breakfast inconsistently [66]. There was also an increased correlation between breakfast consumption and muscle strength [66].

On a school level, every state has different requirements for the frequency and time of physical education classes in school [27]. Even when states have these guidelines, many schools do not comply with the mandates [27]. Several schools need to hire teachers with training in physical education [27]. Students not gaining health literacy through these classes lack a foundation to build future habits [67].

On a community scale, unfortunately, many children do not have access to a safe environment to be more active [37]. These unsafe environments could be determined by limited access to appropriate equipment, vandalized properties, crime levels, substance use, and infrastructure (ex: inadequate lighting) [68]. A literature review found that these factors led to decreased activity and a modest increase in BMI [68]. Policy changes have the potential to focus on these limitations. The American Academy of Pediatrics (AAP) has encouraged support for policies to increase funding for public parks, promote active transit options, promote safe walking routes to schools, and allow for dedicated spaces to walk and bike on streets [69]. The AAP also supports the FIT Kids Act to improve physical activity in schools by supporting the education of teachers and creating regulatory processes [69].

### 1.7. Sleep

A total of 60–70% of American children do not meet the sleep recommendations created by the American Academy of Sleep Medicine and endorsed by the American Academy of Pediatrics, which recommends that teenagers sleep 8–10 h nightly [40,70,71]. In China, this number is even higher, with 91.1% of 16- to 18-year-olds reporting sleep deprivation [72]. However, sleep concerns arise much earlier, with 22% of 9-month-olds experiencing sleep difficulties [73]. Sleep disturbances are also more prevalent in patients diagnosed with autism spectrum disorder and/or attention-deficit/hyperactivity disorder [73]. Adolescents have a phase delay of up to 2 h compared to middle childhood but generally have to wake up earlier for school. This physiologic change impairing sleep duration and quality, as well as societal pressures, including homework and after-school activities, can make it challenging for adolescents to achieve adequate sleep [74,75]. Inadequate sleep can also alter thyroid-stimulating hormone regulation, which can lead to glucose dysregulation and insulin resistance [75]. Some clinical studies have proven that fragmented sleep causes altered glucose metabolism and decreased insulin sensitivity [76]. Many adolescents are exposed to excessive screen time and caffeine, and aspects of the physical environment including noise exposure, screen time, and distractions in the room (i.e., pets and siblings) can hinder sleep quality and duration [74]. Increased screen time can also contribute to poor sleep patterns, reduced feelings of satiety, teeth grinding, and increased consumption of unhealthy snacks [30,73]. One student from the previously referred-to study of Canadian adolescents shared that when playing video games, he would try to go to bed and would have difficulty falling asleep. As a result, he would resume playing games and notes there are periods of time in which games are played for 11–12 h [23]. Other modifiable factors include time utilization between homework, extra-curricular activities, jobs, social activities, and no structured bedtime enforced by parents [74].

Short duration and later sleep times can alter hunger-appetite regulating hormones, such as ghrelin and leptin, leading to sleep disorders and childhood obesity [40,72,75]. A study of Greek fifth and sixth graders identified that children who went to bed after 11 pm on weekdays had up to a 50% higher chance of having overweight or obesity, while children who woke up before 7 am on weekends had up to 100% higher odds of having overweight or obesity [70,77]. This study recognized that children have different sleep schedules on weekdays versus weekends and found that children who experienced decreased sleep duration on weekdays but caught up on weekends were less likely to have overweight or obesity compared to children who had either less or increased sleep on weekdays and weekends [70]. These results have similar outcomes to a study of over 2000 children in Japan and a meta-analysis of five studies ranging from 411 to 16,028 participants in China [72,73]. A different study reviewed by Miller found that bedtimes after 9 pm can increase adiposity independent of sleep duration [78]. This same review found that compared to children who sleep early and wake up early, those who sleep late and wake up late were more likely to have overweight and be less active [78]. These findings were consistent with a study conducted by Skjåkødegård’s team [79]. It has also been proposed that those who wake up later are more likely to skip breakfast, affecting eating patterns for the rest of the day [79]. Students identified that these sleep schedules further shift in the summer when there is less structure created by the school day [23].

While most trials have focused on adults, a randomized crossover trial looking at 37 pediatric patients 8–11 years old showed that compared to decreasing sleep duration by 1.5 h every night, increasing sleep duration by 1.5 h every night over a week led to lower food intake and lower body weight [74]. The consistent driver for weight gain that has been identified is that insufficient sleep increases food intake [74,79]. It is likely that insufficient sleep leading to increased food intake is driven by hedonic rather than hormonal factors [74]. This can be further detailed by identifying that those who sleep an insufficient amount tend to crave more energy-dense foods, which are generally higher in fat and sugar [74].

As pediatric obesity becomes more prevalent, so has the pervasiveness of obstructive sleep apnea. When compared to children who have normal weight, children with overweight or obesity had a 24–61% higher risk of developing sleep apnea [80]. Physiologically, patients with obesity may experience increased fat deposition in the pharyngeal muscles, increased mass effect in the upper airway, decreased chest wall compliance, and a blunting of central respiratory drive [80]. These physiologic changes can increase the risk, as well as the severity, of obstructive sleep apnea. Sleep apnea can result in altered and shorter duration of sleep, which can increase appetite and BMI [80]. Screening and treatment of sleep apnea is important in improving sleep quality in this patient subsect.

Several of the papers reviewed thus far still note that the link between short sleep duration and obesity is still unclear. While mechanisms have been proposed, more research in this field is crucial to further understand the association between obesity and sleep [72,77].

### 1.8. Psychological and Familial Support

Best practice guidelines note the importance of psychosocial factors in the care of youth with obesity, with psychologists and other mental health professionals playing critical roles in assessing and treating eating disorders and facilitating lifestyle change [81]. While promising empirical support exists for the use of cognitive-behavioral interventions for binge and emotional eating, many barriers to care exist, including a paucity of trained providers and long waiting times, particularly outside of metropolitan areas [82]. Furthermore, the cost of these services is often prohibitive, as a lack of parity for mental health services continues to persist [83].

Meta-analyses have found strong evidence for the role of parental support in the effectiveness of family-based interventions for children with overweight or obesity [84]. While parental support can positively influence children’s weight, these efforts may be influenced by friends and other relatives, primarily grandparents, which can interfere with parents’ ability to maintain healthy food habits [29]. For example, a US-based study showed that grandparents often provide sugary and processed treats to bond with grandchildren [85], even when admitting that such choices are unhealthy [86]. A Swedish-based longitudinal study found that the support of siblings, grandparents, teachers, and friends’ parents positively influenced the maintenance of healthy routines for children, even years after obesity treatment [29]. This underscores the influence and importance of environmental factors in addition to parental support, particularly as children grow older.

While parental support is undeniably important in the treatment of pediatric obesity, parental attitudes, and perceptions can interfere with the initiation of treatment. One study found that when children are referred to tertiary centers for further treatment, many parents think care or additional care is needed [87]. In addition to situational factors (time, cost, etc.), parents endorsed a perceived lack of effectiveness and a perceived lack of control over children’s behavior as reasons for failing to utilize specialty centers [87]. Another study investigated the reasons for discontinuing treatment for pediatric obesity at a specialty clinic, which included family factors (perceived lack of progress, children’s lack of motivation, and perceived lack of family support), logistical factors (scheduling, distance, and cost), and health service factors (unmet care needs, limited menu of services, and no perceived need for further support) [88]. More research is needed to investigate and intervene with barriers to initiating and maintaining treatment.

### 1.9. Limited Treatment Options

Initially, pediatric obesity guidelines incorporating pharmacotherapy were established in January 2023 [89]. These guidelines specify the role of pharmacotherapy use in patients 12 years of age and older [89]. The Food and Drug Administration has approved treatments for this age group, including Orlistat, Liraglutide, Semaglutide, Setmelanotide, Phentermine, and Qsymia [89]. There are currently clinical trials studying the use of Semaglutide in patients 6–12 years old for the treatment of obesity (STEP Young trial) and Tirzepatide in patients ages 10–18 years old for the use of type 2 diabetes management (SURPASS-PEDS trial) [90,91]. As research advances, one can expect to see more pharmacological options available for pediatric patients.

Given their efficacy, there has been increased demand for the use of glucagon-like peptide 1 agonists within current treatment options [92]. From an economic perspective, without insurance coverage for these drugs, they currently cost over USD 1000 for a one-month supply [92]. Another barrier to obtaining these medications includes supply chain issues. Since late 2022, unexpected drug shortages for Semaglutide and Dulaglutide have intermittently occurred [93]. This issue has persisted with the approval of Tirzepatide for treating obesity [94]. These barriers make it difficult for patients to initiate and continue treatment reliably.

In addition to pharmacotherapy options, surgical techniques have also been utilized for pediatric patients with severe obesity. Roughly 5% of adolescents meet clinical criteria for metabolic and bariatric surgery [95]. This level of severe obesity with young onset will likely culminate in the progression of numerous related comorbid conditions and shortened life expectancy. Hence, in these cases, it is essential to consider surgical interventions, as the benefits outweigh the risks [89]. Nationally, only 1700 metabolic and bariatric surgeries are performed annually [95]. One barrier is the limited number of treatment centers. Currently, only seven centers in the United States offer surgical options to adolescents [96]. Another limitation includes access to care for minority populations. Currently, approximately 50% of adolescents undergoing surgical intervention are Caucasian, despite Hispanic and Black adolescents being more likely to meet the criteria [95].

### 1.10. Continuity of Care

Ensuring patients can continue care also relies on patients scheduling regular appointments. The most common reasons for missing appointments include forgetting about the appointment, transportation barriers, and parents’ ability to take time off work [31]. Other reasons for high attrition include missing school, the distance to the clinic from home, and children not being ready to change [21]. More pediatric patients are likely to miss well-child visits, often when preventive care, lifestyle counseling, and weight concerns are addressed [31].

## 2. Conclusions

The prevalence of childhood obesity has only continued to expand over the past several years. The long-term consequences are becoming more prevalent, with more children being diagnosed with type 2 diabetes, obstructive sleep apnea, and hypertension than ever before and at an earlier age. While the prevention of obesity remains a key focus, as children continue to develop overweight and obesity, the priority must shift toward aggressive treatment. However, as reviewed here, the barriers to treatment are extensive. These barriers exist on individual and local scales and transcend to community and policy levels.

Addressing obesity requires both individual responsibility and collective action. While traditional approaches have focused on changing individual behavior, effective strategies must also include structural interventions to create healthier environments, as individual choices are heavily influenced by environmental factors. Public health successes in other areas, such as sanitation and immunization, demonstrate the effectiveness of collective action [63].

Obesity is stigmatized in society, policy, and healthcare, reducing health-seeking behaviors and hindering policy improvements. Government responsibility includes educating about weight gain causes and using “nudge” strategies to encourage healthy choices. Shifting the narrative involves understanding that treatments must be offered, without blaming individuals if treatments fail. Obesity should be treated medically, like hypertension, with collective responsibility for resource allocation and patient education. Personalized medicine and acknowledging biological determinants of obesity can reduce stigma and suffering, promoting a more nuanced cultural awareness of the disease [45].

A multidisciplinary focus will be required to address these concerns. Childhood obesity can only begin to be grappled with when patients, families, community members, educators, policymakers, and medical professionals navigate the complexities of this disease state as a unit. Until then, as medical professionals, we must prioritize the individual in front of us and leverage accessible resources for the best interest of our patients.
